# Using Multiparametric Cardiac Magnetic Resonance to Phenotype and Differentiate Biopsy-Proven Chronic from Healed Myocarditis and Dilated Cardiomyopathy

**DOI:** 10.3390/jcm11175047

**Published:** 2022-08-28

**Authors:** Patrick Krumm, Jan M. Brendel, Karin Klingel, Karin A. L. Müller, Jens Kübler, Christoph Gräni, Meinrad Gawaz, Konstantin Nikolaou, Simon Greulich

**Affiliations:** 1 Department of Radiology, Diagnostic and Interventional Radiology, University of Tübingen, Hoppe-Seyler-Straße 3, 72076 Tübingen, Germany; 2 Cardiopathology, Institute for Pathology and Neuropathology, University of Tübingen, Liebermeisterstraße 8, 72076 Tübingen, Germany; 3 Department of Internal Medicine III, Cardiology and Angiology, University of Tübingen, Otfried-Müller-Straße 10, 72076 Tübingen, Germany; 4 Department of Cardiology and Angiology, University of Bern, Freiburgstrasse 18, CH-3010 Bern, Switzerland

**Keywords:** myocarditis, dilated cardiomyopathy, DCM, magnetic resonance imaging, CMR, LGE, T1 mapping, T2 mapping, ECV, endomyocardial biopsy

## Abstract

**(1) Objectives**: To discriminate biopsy-proven myocarditis (chronic vs. healed myocarditis) and to differentiate from dilated cardiomyopathy (DCM) using cardiac magnetic resonance (CMR). **(2) Methods**: A total of 259 consecutive patients (age 51 ± 15 years; 28% female) who underwent both endomyocardial biopsy (EMB) and CMR in the years 2008–2021 were evaluated. According to right-ventricular EMB results, patients were divided into either chronic (*n* = 130, 50%) or healed lymphocytic myocarditis (*n* = 60, 23%) or DCM (*n* = 69, 27%). The CMR protocol included functional, strain, and late gadolinium enhancement (LGE) imaging, T2w imaging, and T2 mapping. **(3) Results**: Left-ventricular ejection fraction (LV-EF) was higher, and the indexed end-diastolic volume (EDV) was lower in myocarditis patients (chronic: 42%, median 96 mL/m²; healed: 49%, 86 mL/m²) compared to the DCM patients (31%, 120 mL/m²), *p* < 0.0001. Strain analysis demonstrated lower contractility in DCM patients vs. myocarditis patients, *p* < 0.0001. Myocarditis patients demonstrated a higher LGE prevalence (68% chronic; 59% healed) than the DCM patients (45%), *p* = 0.01. Chronic myocarditis patients showed a higher myocardial edema prevalence and ratio (59%, median 1.3) than healed myocarditis (23%, 1.3) and DCM patients (13%, 1.0), *p* < 0.0001. T2 mapping revealed elevated values more frequently in chronic (90%) than in healed (21%) myocarditis and DCM (23%), *p* < 0.0001. T2 mapping yielded an AUC of 0.89 (sensitivity 90%, specificity 76%) in the discrimination of chronic from healed myocarditis and an AUC of 0.92 (sensitivity 86%, specificity 91%) in the discrimination of chronic myocarditis from DCM, both *p* < 0.0001. **(4) Conclusions**: Multiparametric CMR imaging, including functional parameters, LGE and T2 mapping, may allow differentiation of chronic from healed myocarditis and DCM and therefore help to optimize patient management in this clinical setting.

## 1. Introduction

Diagnosing myocarditis is challenging due to varying clinical presentation and degrees of myocardial damage ranging from slight functional and morphological alterations to terminal heart failure [1]. In this context, the differentiation of chronic from healed myocarditis and dilated cardiomyopathy (DCM) as a potential end-stage of myocarditis may be difficult, and, hence, clinically relevant [2]. Besides endomyocardial biopsy (EMB) as a reference standard for diagnosis of inflammatory heart diseases, non-invasive cardiac magnetic resonance (CMR) imaging is an emerging method to characterize the myocardium [3,4,5,6] non-invasively.

Various studies have underscored the pivotal role of CMR in confirming clinically suspected myocarditis with functional, T2-weighted (T2w), T1 and T2 mapping and late gadolinium enhancement (LGE) sequences [3,6,7,8]. 

However, there is a lack of data for the discrimination of biopsy-proven chronic from healed myocarditis and DCM. This is of clinical importance since these entities may present with similar clinical signs but might need different treatment and monitoring regimens. 

Therefore, this study aimed to investigate if multiparametric CMR offers reliable non-invasive parameters allowing discrimination of chronic vs. healed myocarditis and DCM.

## 2. Materials and Methods

### 2.1. Study Population

In this single-centre study, 259 consecutive patients (187 patients retrospectively, 72 patients prospectively) with biopsy-proven chronic or healed lymphocytic myocarditis and DCM were enrolled from January 2008 to March 2021. All patients underwent 1.5T CMR imaging. Patients’ symptoms, cardiac risk factors, medication and laboratory values were recorded. The institutional review board approved the study protocol. All prospectively included patients gave written informed consent. The institutional review board waived informed consent for the retrospectively recruited cohort.

### 2.2. Endomyocardial Biopsy Protocol

Endomyocardial biopsies were performed in all patients according to current ESC diagnostic guidelines [4]. At least five biopsies were taken, fixed in 4% phosphate-buffered formaldehyde, and embedded in paraffin. Four µm thick tissue samples were stained with Masson’s trichrome, hematoxylin-eosin as well as Giemsa and examined by light microscopy. 

For immunohistological detection of cardiac immune cells, a monoclonal rabbit-anti-CD3 antibody (Clone SP7, 1:500, Novocastra Laboratories, Newcastle upon Tyne, United Kingdom), a monoclonal mouse anti-human CD68 antibody (Clone PG-M1, 1:50) and a monoclonal mouse anti-human HLA-DR alpha-chain antibody (clone TAL.1B5, 1:50), both from DAKO, Hamburg, Germany, were used. Immunohistochemical analysis was performed on an automated immunostainer following the manufacturer’s protocol (Benchmark; Ventana Medical Systems, Tucson, AZ, USA) and using the ultraView detection system (Ventana) and diaminobenzidine as substrate. Tissue sections were counterstained with hematoxylin. The detection of >14 infiltrating leukocytes/mm² (including >7 CD3+ T-lymphocytes and/or CD68+ macrophages) in the presence of myocyte damage and/or fibrosis in addition to enhanced human leukocyte antigen class II expression in professional antigen-presenting immune cells and endothelium was used for the diagnosis of myocarditis [9].

### 2.3. Definitions of Different Stages of Lymphocytic Myocarditis and DCM

**Figure 1:**
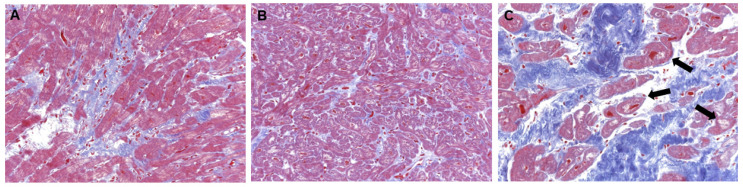
**Histology in different stages of lymphocytic myocarditis and DCM.** EMB samples of patients demonstrating (**A**) chronic myocarditis, (**B**) healed myocarditis and (**C**) DCM. Chronic myocarditis shows focal and/or diffuse fibrosis (blue tissue) but no myocyte necrosis. Healed myocarditis shows no myocyte necrosis and mild interstitial fibrosis. DCM is characterized by hypertrophic and/or atrophic myocytes with loss of myofibrils and vacuolar degeneration (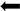 ) as well as severe focal and/or diffuse interstitial fibrosis.

1. *Chronic* myocarditis: No myocyte necrosis, >14 infiltrating leukocytes/mm², focal and/or diffuse fibrosis;
2. *Healed* myocarditis: No myocyte necrosis, mild focal and/or diffuse fibrosis, partial presence of macrophages;
3. DCM: hypertrophic/atrophic myocytes with loss of myofibrils and vacuolar degeneration, severe focal and/or diffuse interstitial fibrosis, no inflammation; also see Figure 1.

### 2.4. Detection of Viral Genomes

Deoxyribonucleic acid and ribonucleic acid were extracted using proteinase-K digestion, followed by extraction with phenol/chloroform. Nested polymerase chain reaction/reverse transcriptase polymerase chain reaction was performed for the detection of parvovirus B19 (PVB19), Epstein–Barr virus (EBV), and human herpesvirus type 6 (HHV6). As a control for the successful extraction of deoxyribonucleic acid and ribonucleic acid, oligonucleotide sequences were chosen from the glyceraldehyde-3-phosphate-dehydrogenase gene. The specificity of all viral amplification products was confirmed by automatic deoxyribonucleic acid sequencing [9].

### 2.5. CMR Image Acquisition

CMR examinations were performed on 1.5T scanners (MAGNETOM Avanto and Aera, SIEMENS Healthcare, Erlangen, Germany). The examination protocol included functional, strain, T2w, T1mapping/T2 mapping and extracellular volume fraction (ECV) calculation (from 2018 on) as well as LGE imaging 10 min after intravenous administration of contrast agent Gadobutrol (Gadovist, Bayer Healthcare, Leverkusen, Germany) with a dosage of 0.15 mmol/kg body weight. Sequence parameters for CMR imaging are given in detail in the Supplementary File S1.

### 2.6. CMR Image Analysis

Image analysis was performed by two experienced CMR readers in consensus using dedicated software (cvi42 Version 5.13, Circle Cardiovascular Imaging, Calgary, AB, Canada) and according to the Society for Cardiovascular Magnetic Resonance (SCMR) recommendations [10,11]. *Morphological parameters* were: planimetry of the atria and measurement of pericardial effusion performed in the end-diastolic phase in 4CV. Left-ventricular and right-ventricular end-diastolic diameters (EDD) were measured in four-chamber view (4CV) and short-axis (SAX), and myocardial thickness in end-diastole was measured in mid-ventricular SAX. *The quantitative functional volumetric* assessment was performed in a stack of SAX slices with semi-automated endocardial and epicardial border contouring, with careful manual re-adjustment, if necessary, and cutoff values according to reference [12]. *Strain analysis* for global longitudinal (GLS) and global radial strain (GRS) was performed using post-processing CMR feature tracking in 4CV. *T2w FSE imaging* was evaluated by segmental signal ratio through myocardial signal intensity (SI) divided by SI of skeletal muscle. T2w SI ratio of 2.0 or higher was considered a positive finding. *LGE imaging* was evaluated qualitatively and semi-quantitatively: LGE patterns (linear vs. patchy) were qualitatively discriminated and localized (septal vs. lateral wall) and assigned to the myocardial segments according to the 17 segment-model of the American Heart Association [13,14]. Semi-quantitative evaluation of LGE mass was performed with a threshold of >2 standard deviations (SD) above the remote myocardium [8].

*T1 and T2 mapping* were considered elevated if segment evaluation was >2 SD above the mean relaxation time of a healthy in-house control group performed at the same 1.5T scanners (T1 > 1053 ms and T2 > 51 ms). *ECV* > 30% was considered pathologically elevated as previously described [15,16,17]. 

### 2.7. Statistical Analysis

Statistical analysis was performed with JMP (Version 16, SAS Institute Inc., Heidelberg, Germany) and SPSS 27 (IBM Corp., Ehningen, Germany). Continuous variables are indicated as median (interquartile range). Categorical data are indicated as frequency (percentage %). The normality of data was checked visually in data plot curves. For group comparison, Kruskal–Wallis and Steel–Dwass (post hoc) tests were performed in continuous, not normally distributed data; the Fisher–Freeman–Halton test, including a closed step-down procedure for multiple group comparisons, was performed in categorical data. Receiver operating characteristic (ROC) curves were generated for comparison of T2 mapping between entities using the method of Delong et al. [18] (MedCalc, Version 18, MedCalc Software Ltd., Ostend, Belgium). The global level of significance *α* was set to 5%. The local level of significance (*α*_loc_) for each test with dependent variables was corrected according to the Bonferroni equation according to *k* = 55 performed comparisons: α_loc_ = α_glob_/*k* = 0.0009.

## 3. Results

### 3.1. Patient Characteristics

Overall, 259 patients (28% females) with a mean age of 51 ± 15 years were included consisting of *n* = 130 (50%) with chronic myocarditis, *n* = 60 (23%) with healed myocarditis, and *n* = 69 with DCM (27%), Table 1. For retrospective inclusion, 796 patients who had undergone EMB between 2008 and 2018 were screened. A total of 360 patients were excluded due to EMB diagnosis other than myocarditis or DCM. Overall, 23 patients with histopathologically proven acute myocarditis were excluded, and 27 were excluded due to inconclusive EMB diagnosis. Furthermore, we excluded 172 patients without CMR and 18 CMR datasets due to incomplete acquisition. Due to a history of coronary artery disease, 9 patients were excluded. In total, 187 patients remained fully evaluable. 

All 259 (100%) patients were symptomatic at the time of diagnostic workup (≥1 symptom): 187 (72%) demonstrated dyspnea, 67 (26%) demonstrated chest pain, 76 (29%) reported fatigue, 41 (16%) exhibited palpitation or tachycardia, and 66 (25%) presented with other symptoms (upper respiratory tract infect infection, edema). Almost half of all patients (*n* = 105, 41%) were NYHA > II, and almost half of the DCM group (*n* = 30, 43%) were ≥NYHA III. Most patients had impaired LV-EF: 110 (85%) in chronic myocarditis, 46 (77%) in healed myocarditis, and all in DCM.

CMR was performed within a median of 30 (IQR 18–60) days after onset of symptoms in chronic myocarditis, 33 (IQR 9–78) days in healed myocarditis, and 182 (IQR 145–221) days in DCM, respectively. EMB was performed a median of 4 (IQR 1–22) days after or prior to CMR. In 199 (77%) patients, CMR was performed prior to EMB, and in 60 (23%) patients, CMR was performed after EMB. In myocarditis patients, Parvovirus B19 was the most common virus type in EMB. T2w imaging was performed in *n* = 109 of 130 (84%) patients with chronic myocarditis, in *n* = 31 of 60 (52%) patients with healed myocarditis and *n* = 47 of 69 (68%) patients with DCM. T2 mapping was performed in *n* = 21 of 130 (16%) patients with chronic myocarditis, in *n* = 29 of 60 (48%) patients with healed myocarditis and *n* = 22 of 69 (32%) patients with DCM.

### 3.2. Different Stages of Myocarditis

Myocardial edema detected by T2 imaging was significantly higher in chronic (90% of patients with elevated T2 relaxation times and 59% with a positive T2w ratio) vs. healed (21% and 23%, respectively) myocarditis, *p* < 0.0001, Table 2. 

In chronic myocarditis, LVEF was lower and pericardial effusion was more frequent compared to healed myocarditis; see Table 2. 

**Figure 2:**
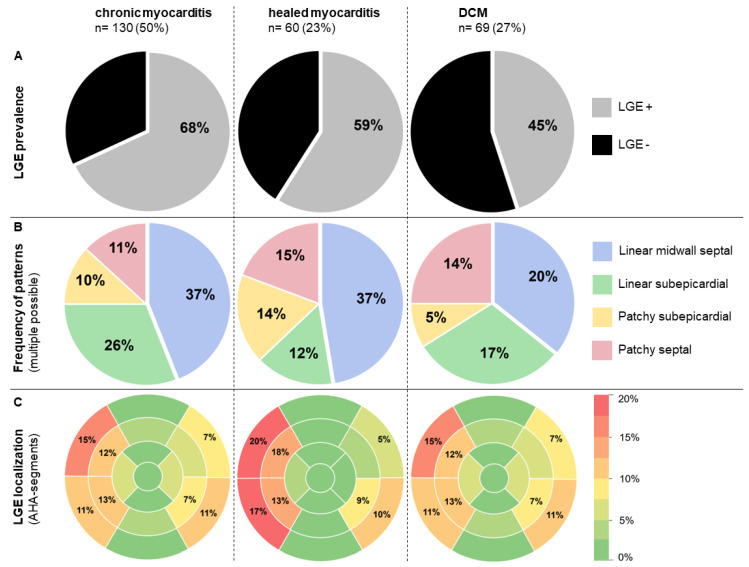
CMR tissue characterization by LGE: overall prevalence and frequency of patterns as well as the frequency of LGE localization per AHA-Segment. (**A**) The prevalence of LGE and (**B**) frequency of various types of LGE patterns in different histological stages of lymphocytic myocarditis (chronic, healed) and DCM. Note the high percentage of linear septal LGE in all groups. (**C**) Distribution of LGE localization (percentage %) using heat-mapped cardiac segmentation models for the different myocarditis stages and DCM. Only LGE patterns with a prevalence ≥5% are numbered.

Both chronic and healed myocarditis demonstrated preferably a linear mid-wall septal LGE pattern (in each entity, 37% of all registered patterns); see Table 2 and Figure 2. In our cohort, we could not find an LGE pattern characteristic of the type of virus infection. LGE CMR parameters in different myocarditis stages are illustrated in Figure 2. 

### 3.3. DCM vs. Chronic and Healed Myocarditis

**Figure 3:**
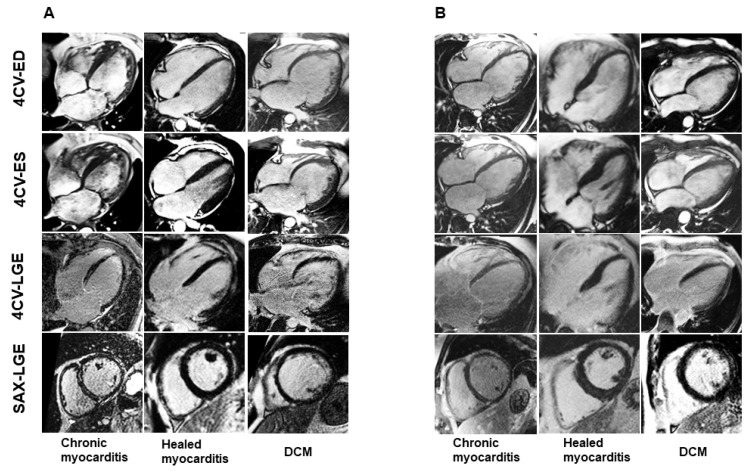
**CMRexamples of chronic healed myocarditis and DCM: different phenotypes.** (**A**) Representative Cine and LGE data of patients with chronic, healed myocarditis and DCM. (**B**) Other examples of chronic, healed myocarditis and DCM show a similar appearance in CMR. DCM: dilated cardiomyopathy, 4CV-ED: end-diastolic four-chamber view, 4CV-ES: end-systolic four-chamber view, 4CV-LGE: late gadolinium enhancement four-chamber view, SAX-LGE: late gadolinium enhancement short axis view.

Functional CMR analysis revealed a significantly higher left ventricular ejection fraction (LV-EF) in myocarditis patients (chronic 42% [29–51]; healed 49% [37–56]) compared to DCM patients (31% [21–38]), *p* < 0.0001. The indexed left ventricular end-diastolic volume (LV-EDVi) was significantly lower in the myocarditis groups (chronic 96 [82–116] ml/m²; healed 86 [75–107] mL/m²) vs. the DCM group (120 [98–150] mL/m²), *p* < 0.0001, as shown in Table 2. Myocardial strain evaluation revealed reduced values in all groups, with a significant difference between myocarditis and DCM patients, *p* < 0.0001: global longitudinal strain (GLS) in chronic myocarditis median −11%, healed myocarditis −13%, DCM −9%; global radial strain (GRS) in chronic myocarditis median 17%, healed myocarditis 20%; DCM 12%, Table 2. LGE prevalence was 68% in chronic myocarditis, 59% in healed myocarditis and 45% in DCM, *p* = 0.010. LGE occurred in a predominantly linear septal pattern in chronic and healed myocarditis (both 37%) vs. DCM (20%), *p* = 0.030 (Figure 2). For clinical CMR images, see Figure 3. 

**Figure 4:**
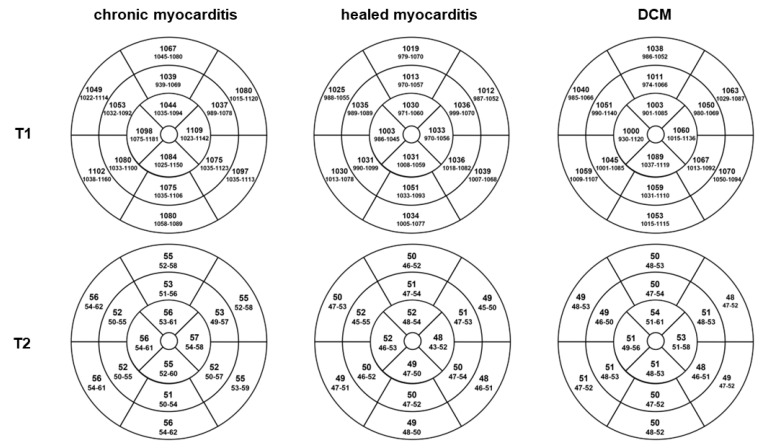
**Mapping results. T1 and T2 relaxation times per AHA segment.** Display of the median values (interquartile range) of T1 and T2 relaxation times in 16 segment models for chronic and healed myocarditis as well as DCM patients. Chronic myocarditis patients had higher T2 relaxation times with a median of 54 ms (IQR 52–57) than healed myocarditis with 50 ms (IQR 46–52) and DCM with 50 ms (IQR 49–52), respectively (*p* < 0.0001). T1 values did not differ significantly.

T2 imaging revealed a higher myocardial edema prevalence in chronic myocarditis (90% T2 mapping and 59% T2w ratio) than in DCM patients (23% and 13%, respectively), *p* < 0.0001. T2 relaxation times were higher in chronic myocarditis patients with a median of 54 ms (IQR 52–57) than in healed myocarditis with 50 ms (IQR 46–52) and DCM patients with 50 ms (IQR 49–52), respectively (*p* < 0.0001). T1 values did not differ significantly, as shown in Figure 4. 

**Figure 5:**
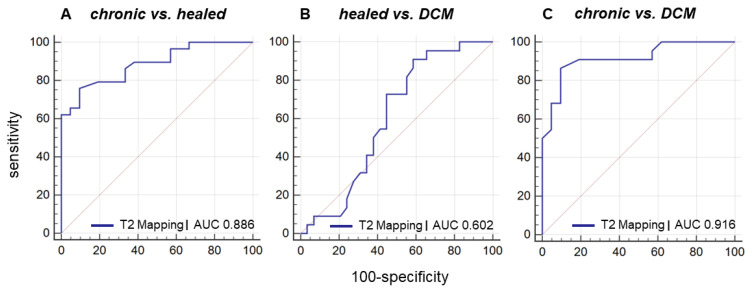
**ROC curve for discrimination of chronic myocarditis from healed myocarditis and DCM using T2 mapping.** Receiver operating characteristic (ROC) curve for discrimination of chronic myocarditis from healed myocarditis and DCM using T2 mapping shows (**A**) an area under the curve (AUC) of 0.886 (sensitivity 90%, specificity 76%) with an associated criterion >51 ms in the discrimination of chronic vs. healed myocarditis (*p* < 0.0001), (**B**) an AUC of 0.602 in the discrimination of healed myocarditis vs. DCM (not significant), (**C**) and an AUC of 0.916 (sensitivity 86%, specificity 91%) with an associated criterion >52 ms in the discrimination of chronic myocarditis vs. DCM (*p* < 0.0001).

In ROC analysis, T2 mapping showed an area under the curve (AUC) of 0.886 (sensitivity 90%, specificity 76%) with an associated criterion >51 ms in the discrimination of chronic vs. healed myocarditis (*p* < 0.0001), an AUC of 0.602 in the discrimination of healed myocarditis vs. DCM (not significant), and an AUC of 0.916 (sensitivity 86%, specificity 91%) with an associated criterion >52 ms in the discrimination of chronic vs. DCM (*p* < 0.0001), respectively (Figure 5). 

**Figure 6:**
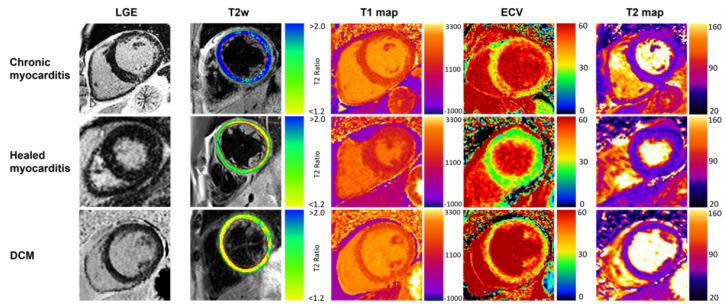
**Typical CMR findings of chronic healed myocarditis and DCM.** Compilation of typical LGE, T2w, T1 and ECV, and T2 mapping appearance in chronic and healed myocarditis plus DCM patients. T2w and T2 mapping show present edema in chronic myocarditis. Increased T1 relaxation times and ECV were present in all groups, prominently depicted in the chronic myocarditis and DCM examples.

For typical CMR findings of chronic and healed myocarditis vs. DCM, see Figure 6. 

## 4. Discussion

This study systematically evaluated non-invasive CMR imaging parameters in the staging of biopsy-proven lymphocytic myocarditis (chronic and healed) and differentiation to DCM. 

Edema in T2 mapping and T2w imaging was significantly higher in chronic myocarditis patients compared to healed myocarditis, underlining the role of T2 imaging as an indicator of myocardial inflammation in myocarditis. DCM patients demonstrated a low prevalence of increased T2 values, underlining the role of T2 as a potential gatekeeper to differentiate chronic from healed myocarditis and DCM. DCM patients showed significantly reduced LV-EF and dilated LV compared to myocarditis patients.

### 4.1. DCM vs. Myocarditis

Heterogeneous groups of myocardial diseases may precede DCM, including autoimmune mechanisms, exposure to toxins, genetic pathogenesis, and infectious agents such as viruses [1,19]. A third of DCM is caused by previous myocarditis [20]. For differentiation in CMR, LV-EF was significantly lower, and LV-EDVi was increased in DCM patients compared to patients with lymphocytic myocarditis, as expected [21]. Myocarditis patients had a higher prevalence of myocardial edema as a surrogate of inflammation. Strain analysis revealed lower values in DCM patients vs. chronic myocarditis patients. However, DCM and all myocarditis groups had reduced strain values, lowering their diagnostic value in a patient group consisting of three different entities of myocardial disease [22]. Hence, differences in strain values between all groups were small with overlaps. However, strain analyses seem to have prognostic implications in myocarditis: Fischer et al. found strain analysis to add prognostic value over clinical values, LV-EF and LGE in myocarditis [23].

LGE prevalence was significantly lower in DCM patients (45%) vs. in chronic (68%) and healed myocarditis (59%). The prevalence of LGE in DCM concurs with previous studies in non-ischemic cardiomyopathies [24,25]. Specifically, a linear mid-wall LGE pattern of the interventricular septum, representing focal replacement fibrosis, can be observed in DCM patients [24]. Likewise, linear mid-wall septal LGE was the most frequently reported LGE pattern in DCM patients in this study. However, a significant number of patients with biopsy-proven healed myocarditis also demonstrated linear mid-wall septal LGE pattern, which is also reported by other CMR studies and seems to be associated with a worse outcome compared to a subepicardial lateral LGE pattern [13,20,26,27]. Focusing exclusively on LGE patterns, DCM may mimic myocarditis and vice versa and should, therefore, not be evaluated independently from other parameters. Additionally, ROC analysis revealed T2 could not differentiate between healed myocarditis vs. DCM for low prevalence in both entities. This underlines the need for a comprehensive CMR approach. 

### 4.2. Chronic vs. Healed Myocarditis

The prevalence of increased T2 was higher in chronic vs. healed myocarditis, suggesting T2 imaging as an indicator of healing in myocarditis. Results of the MyoRacer-Trial support this result, demonstrating edema may well persist in chronic myocarditis [7]. Although problematic on many levels and often prone to artifacts [28,29], T2w images also demonstrated abnormal values in a substantial number of patients. Previous studies have shown the superiority of T2 mapping over T2w images [28,30]. 

Lower native T1 values in our healed myocarditis cohort than in the other groups suggest myocardial healing, making T1 mapping attractive for follow-up in myocarditis patients besides T2 mapping. However, T1 values were not significantly different and did not allow differentiation between chronic vs. healed myocarditis.

Functional, strain, and LGE CMR parameters did not differ significantly between the different histological myocarditis stages (chronic vs. healed) in our cohort. One explanation for some overlap in CMR parameters and clinical data may be a transition from one myocarditis stage to the other (or even to DCM).

Both chronic and healed myocarditis predominantly (37%) demonstrated a linear mid-wall septal LGE, underlining the close relationship between chronic and healed stages in myocarditis. Besides the diagnostic aspect, LGE patterns demonstrate a prognostic value for identifying those patients at the highest risk for adverse events. In addition to inflammation, focal myocardial fibrosis seems to form the substrate for ventricular arrhythmias, with the most unfavorable outcome in the (antero-) septal regions [31]. It would be interesting to investigate if patients with the histological diagnosis of healed myocarditis (implying a favourable clinical course of the patients), who demonstrated the overall highest percentage of mid-wall septal LGE in our cohort, also portend a poor prognosis in the presence of septal mid-wall LGE, or if the negative outcome of this LGE pattern is restricted only to patients with chronic myocarditis or DCM, which has to be demonstrated by another study. 

Nowadays, comprehensive imaging protocols, including T1, T2 and ECV mapping techniques, are recommended for both diagnosis and prognosis of non-ischemic and inflammatory cardiomyopathies [4,8,32]. Unfortunately, mapping techniques were not consistently performed in this study since they were not available most of the enrolment time. Furthermore, T1 mapping is a sensitive but unspecific marker for myocardial damage in both myocarditis and DCM patients [33,34]. Since T1 indicates unspecific myocardial damage, the authors would not expect a reliable accuracy or separation effect for T1 mapping with previously reported overlaps of the groups [34,35]. Further division to inflammatory or fibrotic alterations in T1 can be performed using T2 mapping.

Both T1 mapping and edema imaging techniques have their limitations, and LGE, which we used consistently, is nowadays the most established technique for diagnosis (and prognosis) of myocarditis [9,28,29,36,37]. LGE has a crucial role in risk stratification on outcomes in DCM and myocarditis concerning cardiac adverse events and cardiac remodeling [9,13,26,38,39,40].

Today, EMB is still recommended as the gold standard for diagnosing non-ischemic cardiomyopathies, including myocarditis, as it is the only method to identify the etiology of cardiac inflammation, which is important for the correct therapy of the patient [4]. Nevertheless, to a certain degree, histopathologic workup and definite diagnosis may be (and should be) completed by clinical and other patient information such as ECG, laboratory, CMR and other non-invasive parameters. 

As an outlook, acute or persistent myocardial inflammation may be investigated by a simultaneous hybrid ^18^F-fluorodeoxyglucose positron emission tomography (FDG-PET) CMR as a reference standard for detecting inflammation. The value of PET and whether it may be superior to T2 mapping for detecting myocardial inflammation should be further investigated [41]. 

## 5. Clinical Implications

The clinician might be unable to discriminate between patients with chronic from healed myocarditis and dilated cardiomyopathy since symptoms, echocardiography, ECG and laboratory markers might be similar in all three entities. Yet, this differentiation is of clinical significance for patients since chronic myocarditis and DCM would necessitate more intense monitoring and treatment in addition to regular heart failure therapy. For chronic myocarditis, physical rest as well as abstaining from competitive sports [42,43] might be more beneficial to promote the myocardial healing process than in DCM patients. Moreover, patients with chronic myocarditis and DCM should be carefully monitored by prolonged Holter ECG to record potential (malignant) arrhythmias, and in some of these patients, even an implantable cardioverter-defibrillator might be indicated. Conversely, one might argue that patients with healed myocarditis may have a favorable outcome, which has to be investigated by further studies. Therefore, the exact assignment to one of these three entities may have an impact on both patient treatment and prognosis.

Furthermore, genetic testing may play a role in the development of viral and/or autoimmune myocarditis and a potential progression to DCM [4]. Recently, a current position paper [44] underlined the necessity of a more tailored investigation and management in patients with specific cardiomyopathies, strengthening the role of additional genetic testing, which might have an impact on the patient’s prognosis. Moreover, genetic testing should be considered in all familial forms of myocarditis, in familial cardiomyopathy or when signs of arrhythmogenic cardiomyopathy are present in imaging or electrophysiological tests [45].

## 6. Limitations

As a limitation of this study, a substantial part of the patient cohort has been enrolled retrospectively from 2008 to 2018. Therefore, recent mapping techniques could only be performed on prospectively enrolled patients. Although inferior to T2 mapping, increased T2w values may be present in myocarditis as a sign of edema in persistent inflammation. However, T2w imaging is problematical on many levels [33,46].

EMB samples were taken exclusively from the right ventricle, which might increase a potential sampling error. However, since the distinct histopathological classification was the basis of this study, a sampling error for EMB could not be applied. Performing exclusively right-ventricular biopsy does not represent myocardial alterations of the left ventricle (such as inflammation and fibrosis). However, EMB in areas demonstrating LGE does not necessarily increase the number of positive diagnoses of myocarditis [47].

## 7. Conclusions

Multiparametric CMR imaging, including functional parameters, LGE and T2 mapping, may allow differentiation of chronic from healed myocarditis and DCM and therefore help to optimize patient management in this clinical setting.

## Figures and Tables

**Table 1:** Patient Characteristics.

	Chronic Myocarditis	Healed Myocarditis	DCM	*p*-Value All Groups	*p*-Value Chronic vs. Healed Myocarditis	*p*-Value Chronic Myocarditis vs. DCM	*p*-Value Healed Myocarditis vs. DCM
	*n* = 130 (50%)	*n* = 60 (23%)	*n* = 69 (27%)				
Female	39 (30%)	16 (27%)	17 (25%)	-	-	-	-
Age (yrs)	51 [38–63]	51 [31–65]	54 [46–60]	n.s.	-	-	-
BMI (kg/m²)	26 [24–30]	26 [23–29]	27 [24–31]	n.s.	-	-	-
**CVRF**							
Arterial hypertension	53 (41%)	12 (20%)	32 (46%)	0.009	0.011	n.s.	n.s.
Diabetes	25 (19%)	3 (5%)	10 (14%)	0.031	0.018	n.s.	n.s.
Dyslipidemia	31 (24%)	5 (8%)	11 (16%)	0.036	0.028	n.s.	n.s.
Smoking	34 (26%)	9 (15%)	21 (29%)	n.s.	-	-	-
Family history of CVD	26 (20%)	4 (7%)	6 (9%)	0.019	0.031	0.039	n.s.
Obesity	37 (28%)	9 (15%)	20 (29%)	n.s.	-	-	-
**EMB analysis**							
EBV	2 (2%)	1 (2%)	1 (1%)	n.s.	-	-	-
PVB19	29 (22%)	14 (23%)	2 (3%)	<0.001	n.s.	0.001	<0.001
HHV6	19 (15%)	0	1 (1%)	<0.001	0.004	0.009	n.s.
**Medication**							
Beta blockers	67 (52%)	33 (55%)	48 (70%)	0.046	n.s.	0.023	n.s.
AT1 receptor blockers	17 (13%)	14 (23%)	24 (35%)	0.002	n.s.	<0.001	n.s.
ACE inhibitors	53 (41%)	16 (27%)	31 (45%)	n.s.	-	-	-
Calcium channel blockers	6 (5%)	5 (8%)	7 (10%)	n.s.	-	-	-
Diuretics	39 (30%)	17 (28%)	30 (43%)	n.s.	-	-	-
Aldosterone receptor antagonists	42 (32%)	20 (33%)	38 (55%)	0.007	n.s.	0.002	n.s.
**NYHA-Classification**							
NYHA I	32 (25%)	25 (42%)	15 (22%)	n.s.	-	-	-
NYHA II	37 (28%)	21 (35%)	24 (35%)	n.s.	-	-	-
NYHA III	48 (37%)	10 (17%)	26 (38%)	n.s.	-	-	-
NYHA IV	13 (10%)	4 (7%)	4 (6%)	n.s.	-	-	-
Troponin elevated (>57 ng/L)	5 (4%)	4 (7%)	1 (1%)	n.s.	-	-	-
NT-proBNP elevated (>300 ng/L)	34 (26%)	9 (15%)	24 (35%)	n.s.	-	-	-

Values are given as frequency (percentage %) or median (interquartile range); *p*-values < 0.05 were considered as significant; n.s. = not significant; DCM = dilated cardiomyopathy; BMI = body mass index; CVRF = cardiovascular risk factors; CVD = cardiovascular disease; EMB = endomyocardial biopsy; EBV = Epstein–Barr virus; PVB19 = Parvovirus B19; HHV6 = human herpesvirus 6; AT1 receptor = angiotensin II receptor type 1; ACE = angiotensin converting enzyme; NYHA = New York Heart Association; NT-proBNP = N-terminal pro-B-type natriuretic peptide.

**Table 2:** CMR Results in Chronic and Healed Myocarditis as well as DCM.

	Chronic Myocarditis *n* = 130 (50%)	Healed Myocarditis *n* = 60 (23%)	DCM *n* = 69 (27%)	*p*-Value All Groups	*p*-Value Chronic vs. Healed Myocarditis	*p*-Value Chronic Myocarditis vs. DCM	*p*-Value Healed Myocarditis vs. DCM
**Left atrium**							
Area (cm²) 4CV	25 (21–33)	23 (19–31)	28 (23–37)	0.009	n.s.	n.s.	0.007
**Right atrium**							
Area (cm²) 4CV	25 (22–29)	25 (20–28)	25 (20–32)	n.s.	-	-	-
**Pericardial effusion**							
>5 mm	41 (32%)	6 (10%)	9 (13%)	<0.001	0.002	0.006	n.s.
**Morphology (LV)**							
IVS (mm)	10 (8–11)	10 (8–11)	9 (8–11)	n.s.	-	-	-
LV mass (g)	89 (79–105)	91 (74–124)	99 (78–125)	n.s.	-	-	-
Indexed LV mass (g/m²)	45 (39–53)	48 (36–60)	47 (38–57)	n.s.	-	-	-
**Volumetry (LV)**							
EF (%)	42 (29–51)	49 (37–56)	31 (21–38)	<0.0001	0.018	<0.0001	<0.0001
SV (mL)	77 (60–93)	83 (64–92)	72 (58–91)	n.s.	-	-	-
Indexed SV (mL/m²)	38 (31–45)	42 (35–48)	36 (29–44)	n.s.	-	-	-
EDV (mL)	190 (158–251)	174 (145–204)	243 (195–318)	<0.0001	n.s.	<0.0001	<0.0001
Indexed EDV (mL/m²)	96 (82–116)	86 (75–107)	120 (98–150)	<0.0001	n.s.	<0.0001	<0.0001
ESV (mL)	113 (80–170)	86 (71–114)	173 (123–232)	<0.0001	0.029	<0.0001	<0.0001
Indexed ESV (mL/m²)	56 (40–82)	45 (34–57)	87 (59–110)	<0.0001	n.s.	<0.0001	<0.0001
**Feature tracking (LV)** **Peak strain (%)**							
Global longitudinal	−11 (−14 to −7)	−13 (−15 to −9)	−9 (−12 to −6)	<0.0001	n.s.	0.0007	0.0002
Global radial	17 (11–24)	20 (13–25)	12 (8–17)	<0.0001	n.s.	0.0005	0.0001
**LGE**							
Prevalence	88 (68%)	35 (59%)	31 (45%)	0.010	n.s.	0.003	n.s.
(% of LVMM)	4 (2–6)	3 (2–5)	3 (2–5)	0.003	0.008	0.031	n.s.
**LGE pattern** (multiple possible)							
Linear midwall septal	48 (37%)	22 (37%)	14 (20%)	0.030	n.s.	0.015	0.04
Linear subepicardial	34 (26%)	7 (12%)	12 (17%)	n.s.	-	-	-
Patchy subepicardial	13 (10%)	8 (14%)	3 (5%)	n.s.	-	-	-
Patchy septal	14 (11%)	9 (15%)	10 (14%)	n.s.	-	-	-
**T2w imaging**	***n* = 109 (84%)**	***n* = 31 (52%)**	***n* = 47 (68%)**				
T2 Signal-Ratio entire slice	1.3 (1.1–1.9)	1.3 (1.0–1.5)	1.0 (0.6–1.2)	<0.0001	n.s.	<0.0001	0.005
T2 positive in ≥1 segment	64 (59%)	7 (23%)	6 (13%)	<0.0001	<0.0001	<0.0001	n.s.
**Mapping**	***n* = 21 (16%)**	***n* = 29 (48%)**	***n* = 22 (32%)**				
T1	1063 (1014–1102)	1038 (1010–1066)	1055 (1000–1078)	n.s.	-	-	-
T1 elevated (>1053 ms) *	13 (62%)	9 (31%)	11 (50%)	n.s.	-	-	-
ECV	32 (26–35)	28 (26–33)	30 (26–35)	n.s.	-	-	-
ECV elevated (>30%)	10 (48%)	6 (21%)	8 (36%)	n.s.	-	-	-
T2	54 (52–57)	50 (46–52)	50 (49–52)	<0.0001	<0.0001	<0.0001	n.s.
T2 elevated in ≥1 segment (>51 ms) *	19 (90%)	6 (21%)	5 (23%)	<0.0001	<0.0001	<0.0001	n.s.

Values are given as frequency (percentage %) or median (interquartile range); *p*-values < 0.05 were considered as significant; n.s. = not significant; indexed data are normalized to body surface area; DCM = dilated cardiomyopathy; 4CV = 4-chamber view; LV = left ventricular; IVS = interventricular septum; EF = ejection fraction; SV = stroke volume; EDV = end-diastolic volume; ESV = end-systolic volume; LGE = late gadolinium enhancement; LVMM = left ventricular myocardial mass; ECV = extracellular volume fraction; * > 2SD of control group.

## Data Availability

The datasets analyzed in our study are available from the corresponding author upon reasonable request.

## Supplementary Materials

The following supporting information can be downloaded at: https://www.mdpi.com/article/10.3390/jcm11175047/s1, Supplementary File S1. CMR sequence parameters.
