# Supplementary material for: Using Multiparametric Cardiac Magnetic Resonance to Phenotype and Differentiate Biopsy-Proven Chronic from Healed Myocarditis and Dilated Cardiomyopathy
Source: J Clin Med. 2022 Aug 28;11(17):5047. doi: 10.3390/jcm11175047 (PMC9457265; doi:10.3390/jcm11175047)
Supplement: Supplementary file 1 [file jcm-11-05047-s001.zip › jcm-1841608-supplementary-1.pdf]

Supplementary File S1

To Materials and Methods: CMR image acquisition

All images were acquired in breath hold technique with ECG triggering. Field of View was minimized and adapted to patient's size respectively.

Sequence parameters for functional imaging:

Steady State Free Precession (SSFP) cine loops (repetition time (TR) 3 ms; echo time (TE) 1.5 ms; flip angle 60°, slice thickness 5 mm; matrix 256x192; parallel imaging Generalized Auto-calibrating Partially Parallel Acquisitions (GRAPPA) factor 2; retrospective triggering, 25 frames reconstructed per cardiac cycle) were acquired in standard angulations: four-chamber view (4CV), two-chamber view (2CV) and a stack of short-axis (SAX) slices (gap 5 mm) covering both entire ventricles from base to apex.

Sequence parameters for T2 imaging:

T2-weighted (T2w) FSE sequence was used with TR 719 ms, TE of 76 ms, Trigger pulse 2, Matrix 256 x 159, spectral fat saturation, GRAPPA factor 2, acquisition in 4CV, 2CV and three representative SAX.

For myocardial T2 mapping a product type T2 prepared SSFP sequence (MyoMaps, SIEMENS Healthcare) with T2 preparation pulses at 1) none, 2) 24 ms, 3) 55 ms was acquired in basal, mid-ventricular and apical slice. TR 307.5ms, TE 1.17ms, flip angle 20°, slice thickness 8.0mm, baseline matrix 144 x 192.

Sequence parameters for LGE imaging:

2D inversion recovery (IR) gradient recovery echo (GRE) sequence, TR 11 ms, TE 4.4 ms, flip angle 30°, slice thickness 6 mm, baseline matrix 256. The inversion time was adjusted individually to 260-340 ms, to minimize signal from normal myocardium.

Sequence parameters for T1 and ECV mapping:

A T1 mapping MOLLI sequence (MyoMaps, SIEMENS Healthcare) with acquisition scheme 5(3)3 (vendor label T1 long) was acquired native and 15-20min post contrast after Late Gadolinium Enhancement imaging (LGE). Sequence Type Steady State Free Precession (SSFP).

For RR-interval >700ms: TR 280.6ms, TE 1.12ms, flip angle 35°, slice thickness 8.0mm, baseline matrix 169 x 256.

For RR-interval <700ms: TR 360.6 ms, TE 1.12 ms, flip angle 35°, slice thickness 8.0mm, baseline matrix 169 x 256.
